# Screening of Key Components for Melanogenesis Inhibition of *Polygonum cuspidatum* Extract Based on the Spectrum–Effect Relationship and Molecular Docking

**DOI:** 10.3390/molecules29040857

**Published:** 2024-02-15

**Authors:** Ruojun Du, Lichun Ye, Xinyan Chen, Yan Meng, Lei Zhou, Qiao Chen, Guohua Zheng, Junjie Hu, Zhaohua Shi

**Affiliations:** 1 College of Pharmacy, Hubei University of Chinese Medicine, Wuhan 430065, China; drj_20231211@163.com (R.D.); chenxy_328@163.com (X.C.); yanmeng2016@126.com (Y.M.); zhouyichen20231211@163.com (L.Z.); 18186549240@163.com (Q.C.); 2 Clinical College of Chinese Medicine, Hubei University of Traditional Chinese Medicine, Wuhan 430065, China; lichunye05@hbtcm.edu.cn; 3 Key Laboratory of Resources and Compound of Traditional Chinese Medicine, Ministry of Education, Hubei University of Traditional Chinese Medicine, Wuhan 430065, China; 4 Hubei Shizhen Laboratory, Wuhan 430065, China

**Keywords:** *Polygonum cuspidatum* (PC), tyrosinase (TYR), melanogenesis, microphthalmia-associated transcription factor (MITF), tyrosinase-related protein-1 (TYRP-1), dopachrome tautomerase (DCT)

## Abstract

*Polygonum cuspidatum* (PC) extract has been listed in the “Catalog of Used Cosmetic Ingredients (2021 Edition)”, which can inhibit melanogenesis, thus exerting a whitening effect, and has been widely used in cosmetics. However, there are currently no quality standards for PC extract used in cosmetics, and the bioactive components associated with anti-melanogenesis remain unclear. In view of this, the present study was the first to investigate the spectrum-effect relationship between fingerprints of PC extract and melanogenesis inhibition. Ten batches of PC extract fingerprints were established by HPLC. Pearson’s correlation analysis, gray correlation analysis (GRA) and orthogonal partial least squares regression analysis (OPLSR) were used to screen out resveratrol, emodin and physcion as the main whitening active ingredients using the inhibition of tyrosinase in B16F10 cells as the pharmacological index. Then, the melanogenesis inhibitory effects of the above three components were verified by tyrosinase inhibition and a melanin content assay in B16F10 cells. The interaction between small molecules and proteins was investigated by the molecular docking method, and it was confirmed by quantitative real-time PCR (qRT-PCR) that resveratrol, emodin and physcion significantly down-regulated the transcript levels of melanogenesis-related factors. In conclusion, this study established a general model combining HPLC fingerprinting and melanogenesis inhibition and also analyzed the spectrum–effect relationship of PC extract, which provided theoretical support for the quality control of PC extract in whitening cosmetics.

## 1. Introduction

Melanocytes in the basal layer of the human epidermis are the site of melanin production. After melanin production, it passes from the dendritic tips of melanocytes to the stratum corneum, where it will be excreted with the metabolism of stratum corneum cells [1]. In human skin, melanogenesis is triggered by exposure to UV radiation, resulting in darkening or hyperpigmentation. Excessive melanin accumulation leads to chloasma and other skin diseases, so reducing melanin synthesis is the main method to whiten spots [2]. In mammals, specific melanogenic enzymes that contribute to melanogenesis have been elucidated, such as TYR, TYRP-1, and DCT [3]. TYR has a key rate-limiting activity in the oxidative reactions of melanogenesis and controls two rate-limiting steps: the conversion of L-tyrosine to L-DOPA (as a hydroxylase) and the conversion of DOPA to dopaquinone (as an oxidizing enzyme) [4,5]. Therefore, down-regulation of tyrosinase is the predominant approach for the development of melanogenesis inhibitors. Traditional whitening agents including lead powder, heavy metals and hydroquinone are banned due to their toxicity and irreversible damage to the skin [6]. At present, commercial whitening products such as hydroquinone, kojic acid and arbutin have been used to control the excessive biosynthesis of melanin. However, the long-term use of these products can affect human health [7]. Studies have found that some plant extracts and phytochemicals have significant melanin-inhibitory effects [8,9]. Therefore, using plant extracts to develop natural and safe melanin inhibitors has become a new research trend. Through the advertising sector, we learned that numerous domestic and international brands have introduced cosmetics with natural components, such as the edible skin care products introduced by suitably Bencao and the red cloisonne roots series by French Lancome [10]. We deduced from these findings that natural herbal extracts should have bright research possibilities in the current cosmetic sector.

*Polygonum cuspidatum* (PC) extract has been included in the “Catalog of Used Cosmetic Ingredients (2021 Edition)” and is added to thousands of cosmetic products (https://www.bevol.cn/index.html) (accessed on 25 September 2023). Some studies have shown that PC extracts prepared by different methods have certain TYR inhibitory effects [11,12,13]. Although PC extract is widely used in whitening cosmetics, there are no quality standards for its application in cosmetics. PC extract is rich in resveratrol, a compound that has been the subject of popular research in recent years and has been listed as one of the “100 hottest and most effective anti-aging substances” in the “anti-aging canon” for its whitening, anti-aging and moisturizing properties [14]. Only resveratrol content is used as a quality control indicator for PC extract in the market. However, some ingredients such as stilbenes, flavonoids and anthraquinones have been reported in the literature to inhibit TYR activity to some extent, thereby inhibiting melanogenesis [15,16]. Studies on the material foundation closely associated with melanogenesis inhibition are uncommon, although many studies have demonstrated the melanin-inhibitory effect of PC extract. Therefore, it is crucial to develop quality standards for PC extract in cosmetics and to determine the material basis for the inhibition of melanogenesis by PC extracts.

Spectrum-effect relationship analysis integrates chemical profiles and pharmacological activities to identify the major active ingredients in complex samples. This method has been successfully applied to reveal the material basis of traditional Chinese medicines (TCM) [17]. The spectrum-effect relationship of TCM adopts the combination of the “spectrum” of the chemical composition of TCM and the “effect” of the clinical action of TCM. Among them, the “spectrum” is mostly a fingerprint or feature atlas with holistic and fuzzy features; the “effect” is mostly an indicator that can represent the efficacy of TCM, which should have classical, parsimonious and sensitive features [18,19]. A simple chemical fingerprint cannot effectively assess the relationship between components and TCM efficacy, even though fingerprinting is a widely acknowledged quality evaluation approach of TCM in China. Therefore, it is one of the effective methods to predict and screen the active ingredients of TCM by combining mathematical and statistical methods. Molecular docking is a method of drug design based on the properties of the receptor and the interactions between the receptor and the drug molecule. It is a theoretical simulation of interactions between ligands and receptors, and it predicts their binding modes and affinities [20,21]. Nowadays, molecular docking has become an important technique in the field of computer-aided drug development. As a result, the screening of active ingredients was jointly realized by the combination of spectrum–effect relationship and molecular docking.

In this study, the TYR inhibitory effect of PC extract was evaluated and a fingerprint was performed by HPLC. Then, based on Pearson’s correlation analysis, GRA and OPLSR, the key active components of PC extract with melanogenesis inhibitory effects were identified. Subsequently, the predicted results were verified by in vitro pharmacologic tests. Finally, the molecular docking method was used to predict the interactions between the active ingredients and important targets related to melanogenesis. Meanwhile, the transcript levels of melanogenesis-related factors were confirmed using qRT-PCR. The results of this study will provide an effective basis for the rational application of PC extracts in cosmetics, especially for the development of quality standards for cosmetic ingredients.

## 2. Results

### 2.1. HPLC Fingerprint of 10 Batches of PC Extract

#### 2.1.1. Analysis of the Chromatographic Fingerprint and Similarities

**Figure 1:**
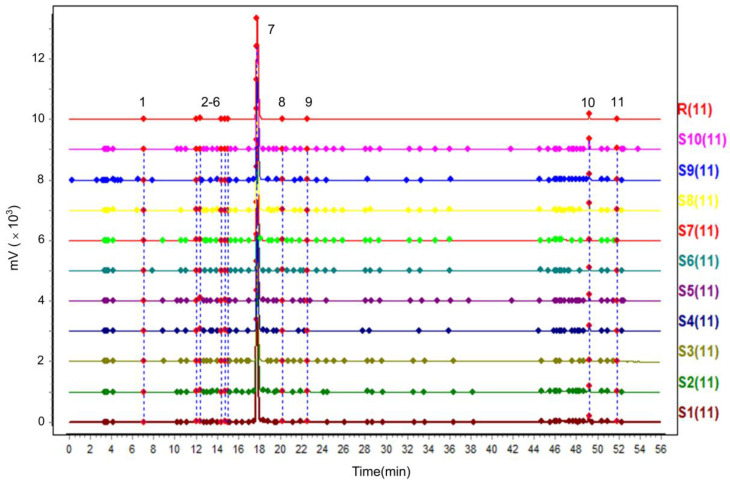
Chromatography fingerprint of 10 batches of PC extract.

As shown in Figure 1, the HPLC fingerprint of 10 batches of PC extract generated a total of 11 common peaks, but the peak areas of the common peaks were different. The similarity of the 10 batches of PC extract was calculated using the control fingerprint profile as a reference, and the similarity values of the 10 batches of PC extract ranged from 0.999 to 1.000.

#### 2.1.2. Method Validation of Fingerprint Analysis

In the reproducibility, precision and stability investigation, the RSD values of relative retention time and peak area of each peak in the sample were less than 3.00% with resveratrol as the reference peak (Table 1). This result indicated that the fingerprint analysis method had good reproducibility, precision and stability, and could be used to establish the fingerprint profile of the PC extract.

### 2.2. Effect of Different Extracts on Whitening Activity

#### 2.2.1. Cell Viability Evaluation of Different Extracts

**Figure 2:**
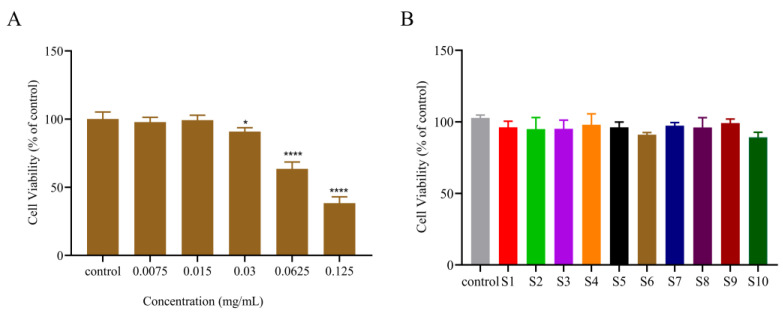
Cytotoxicity of PC extract on B16-F10 cells. (**A**) The effects of PC extract (S6) on B16-F10 cell viability after treatment with different concentrations. (**B**) 10 batches of PC extract from different sources on B16-F10 cell viability after treatment with 0.03 mg/mL concentration. (Compared with control, * *p* < 0.05, **** *p* < 0.0001.)

To determine the optimal cellular administration concentration for TYR inhibition, one batch of PC extract (S6) was randomly selected for the cellular activity evaluation experiments at different concentrations. As can be seen in Figure 2, the cellular activity remained above 85% when the concentration of PC extract was 0.03 mg/mL and below. At the same time, at a concentration of 0.03 mg/mL, the cell activity of ten batches of PC extract remained above 85% after acting on cells. Therefore, 0.03 mg/mL was determined to be the highest safe concentration that the cells can tolerate.

#### 2.2.2. The Inhibition Effect of Different Extracts on Intracellular TYR

**Figure 3:**
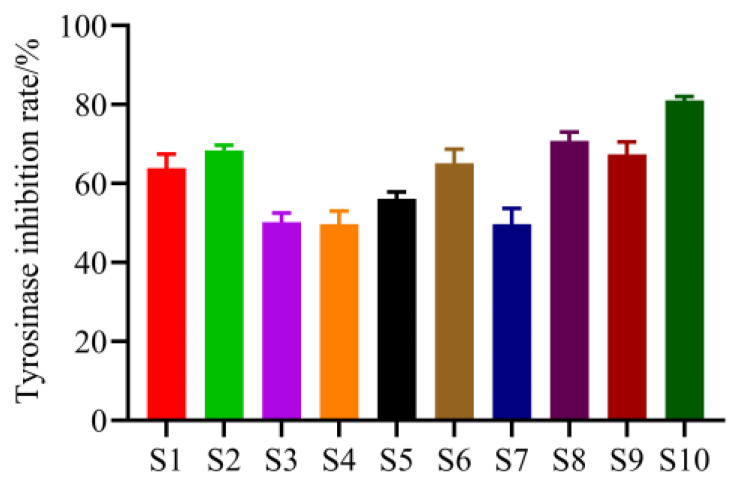
Inhibition rate of tyrosinase in B16–F10 cells after treatment with 10 batches of PC extract.

As shown in Figure 3, 10 batches of PC extract revealed different inhibitory effects on intracellular TYR in B16F10 cells, and the contents of the 10 batches of PC extract varied. As a result, we hypothesized that variations in component content could account for the various TYR inhibition rates. The TYR inhibitory effect of PC extract had an unclear material basis, and this area of study was lacking. In order to employ PC extract in whitening cosmetics, it was necessary to screen possible chemicals that were either directly or indirectly related to the important TYR inhibitory function.

### 2.3. Analysis of Spectrum–Effect Relationships

#### 2.3.1. Pearson’s Correlation Analysis

Pearson’s correlation analysis result of the common peak areas from 10 batches of PC extract and their TYR inhibition effect was shown in Table 2. The positive Pearson’s correlation coefficient indicates a certain degree of positivity, and the larger the Pearson’s correlation coefficient, the more significant the correlation. It can be found that the Pearson’s correlation coefficients of peak 7, peak 10 and peak 11 were larger than those of other common peaks, significantly different from other common peaks (*p* ≤ 0.05), indicating that the three components represented by these three peaks could emerge obvious impact on TYR activity.

#### 2.3.2. Gray Correlation Analysis (GRA)

According to the GRA method, the correlation values and correlation ranking of 11 common peaks were exhibited in Table 3. Since the correlation values of P7, P8, P4, P5 and P10 were all greater than 0.8, it can be inferred that the active ingredients represented by these 5 common peaks could have a stronger relevance with the TYR inhibition effect. By comparison, the correlation value of peak 6 was less than 0.6, indicating a weak relevance with the TYR inhibition effect [22]. As seen in Table 3, the correlation from high to low was P7 > P8 > P4 > P5 > P10 > P2 > P11 > P9 > P1 > P3 > P6.

#### 2.3.3. Orthogonal Partial Least Squares Regression Analysis (OPLSR)

**Figure 4:**
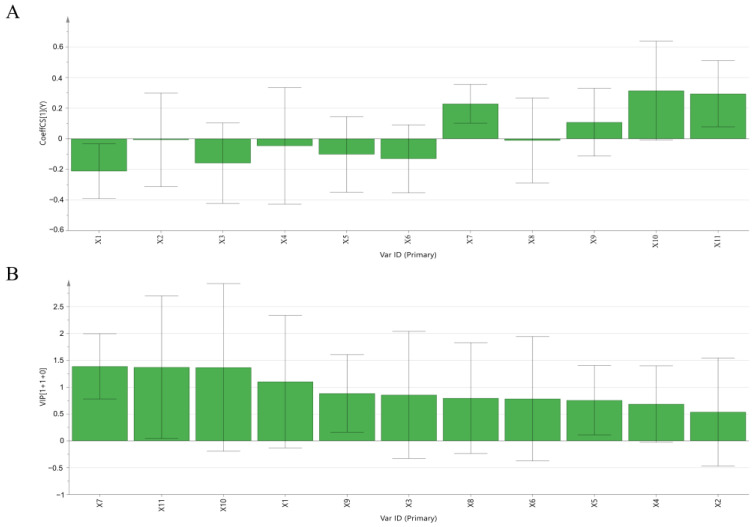
Establishment of spectrum-effect relationship by the OPLSR model. (**A**) Correlation coefficients and (**B**) VIP values.

The established regression equation was Y = 5.8961 − 0.2119X_1_ − 0.0073X_2_ − 0.1595X_3_ − 0.0464X_4_ − 0.1022X_5_ − 0.1308X_6_ + 0.2285X_7_ − 0.0116X_8_ + 0.1082X_9_ + 0.3142X_10_ + 0.2939X_11_, and the model diagnostic parameters R^2^X = 0.585, R^2^Y = 0.911, Q^2^ = 0.742, indicating that the regression model had strong fitting explanatory power and model predictive power. As shown in Figure 4A, the positive regression coefficients for peaks 7, 9, 10 and 11 indicated that these peaks are positively correlated with activity.

The variable importance (VIP value) can reflect the importance of the X variable and its ability to explain the Y variable, while the variables with a VIP value greater than 1 are considered to have a more important ability than variables with a VIP value less than 1 [23]. As shown in Figure 4B, the VIP values of P7, P11, P10, and P1 were all greater than 1. Therefore, P7, P11, and P10 were highly and positively correlated with efficacy.

#### 2.3.4. Comprehensive Statistical Analysis

**Figure 5:**
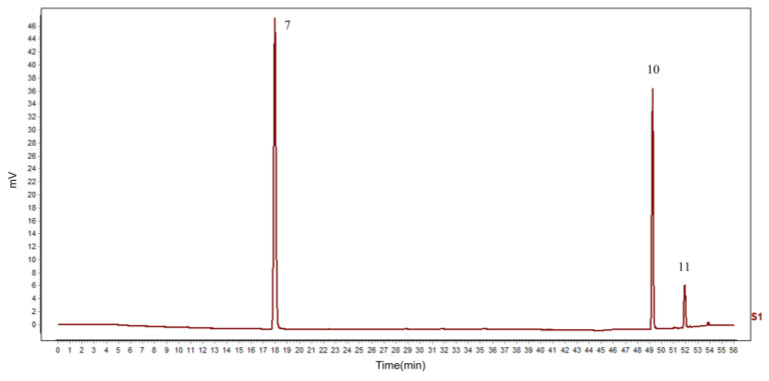
HPLC chromatograms of the mixed standard solution (7: resveratrol; 10: emodin; 11: physcion).

Combined with the results of bivariate correlation analysis, gray analysis and OPLSR analysis, it can be easily inferred that peaks 7, 10 and 11 could be important in PC extract evidently correlated with TYR activity. After comparing the standard products, we found that these three peaks belong to the three bioactive compounds resveratrol, emodin and physcion, respectively. The chromatogram of the standards is shown in Figure 5.

### 2.4. Activity Verification of Effect-Related Compounds

#### 2.4.1. In Vitro Cytotoxicity of Effect-Related Components on B16F10 Cells

**Figure 6:**
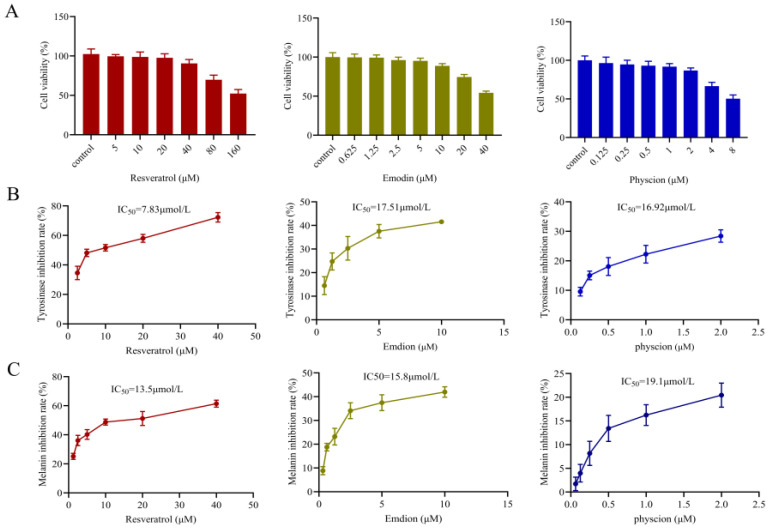
Inhibition effects of three components (resveratrol, emodin and physcion) on tyrosinase and melanogenesis in B16F10 cells. (**A**) In vitro cytotoxicity of three components (resveratrol, emodin and physcion) on cultured B16F10 cells. (**B**) The inhibitory effect of resveratrol, emodin and physcion on tyrosinase inhibition in B16F10 cells was determined. (**C**) The inhibition effect of cellular melanogenesis was determined.

The CCK-8 assay was adopted to detect the cytotoxicity of the three active components on B16F10 cells. As shown in Figure 6A, a dose-dependent inhibition effect on cell viability was observed after incubation with different concentrations of resveratrol, emodin and physcion for 48 h. When the administration concentrations of resveratrol, emodin and physcion were as high as 40 μM, 10 μM and 2.5 μM, respectively, more than 85% of cell viability still survived. Therefore, 40 μM, 10 μM and 2.5 μM was set as the maximum concentrations for resveratrol, emodin and physcion in subsequent B16F10 cells experiments, respectively.

#### 2.4.2. TYR Activity and Melanogenesis

As shown in Figure 6B, TYR inhibition rates of resveratrol, emodin and physcion increased with increasing concentration, and the IC_50_ values of TYR inhibition rate by the three components were 7.83 μM, 17.51 μM, and 16.52 μM, respectively. These data suggested that the three components all had a certain TYR inhibitory effect. Melanin inhibition results of resveratrol, emodin and physcion were shown in Figure 6C. The IC_50_ values of melanin content were 13.5 μM, 15.80 μM, and 19.09 μM, respectively. These data indicated that all three predictive components were effective in inhibiting melanin production and had a certain whitening potential.

### 2.5. The Regulation Mechanism of Melanogenesis in B16F10 Cells

#### 2.5.1. Molecular Docking Simulations of Three Components Interaction with TYR, TYRP-1, DCT and MITF

**Figure 7:**
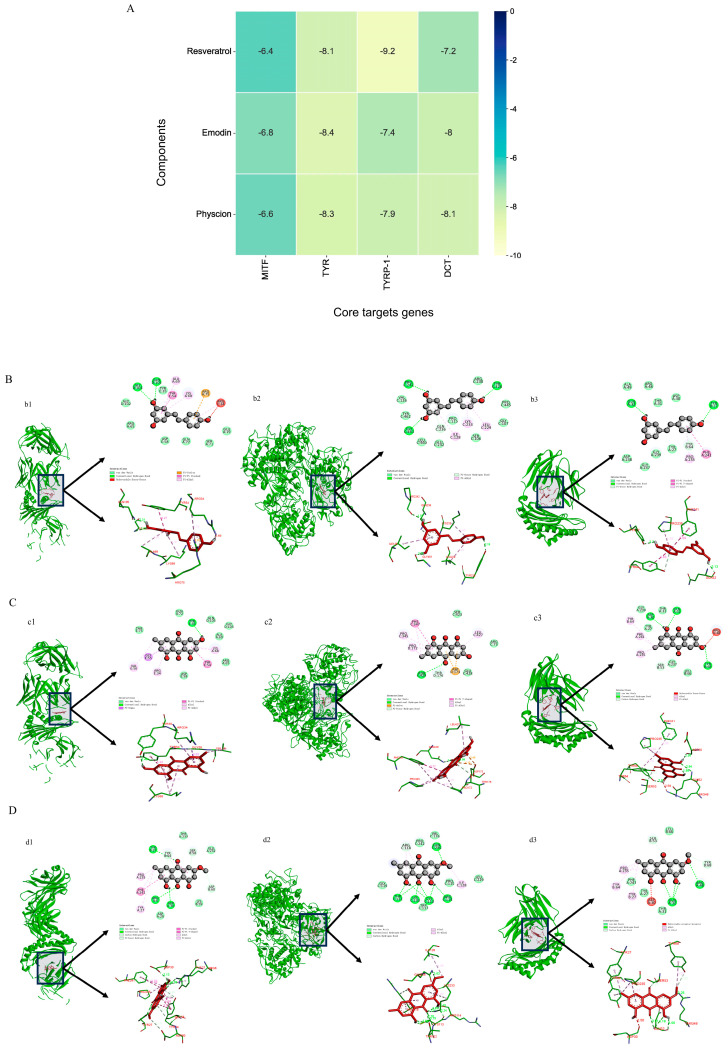
Molecular docking simulation of interactions between MITF, TYR, TYRP-1, DCT and compounds. (**A**) Heat map of binding energies; (**B**) the 3D and 2D diagram of the docking between resveratrol and TYR (**b1**), TYRP-1 (**b2**), and DCT (**b3**); (**C**) the 3D and 2D diagram of the docking between emodin and TYR (**c1**), TYRP-1 (**c2**), and DCT (**c3**); (**D**) the 3D and 2D diagram of the docking between physcion and TYR (**d1**), TYRP-1 (**d2**), and DCT (**d3**).

To further determine the binding mode, molecular docking technology was used to analyze the binding of the active ingredient to key receptors. Molecular docking is performed to identify the potential interactions of resveratrol, emodin, and physcion with TYR, MITF, TYRP-1 and DCT, and docking energy is the parameter used to estimate the interaction between the ligand and its receptor, the more negative the value of binding energy, the stronger the binding effect. As shown in Figure 7A, the binding energy values of the three components with TYR, MITF, TYRP-1 and DCT were all below −5.0 kcal/mol, indicating good binding activity between them. At the same time, the binding energy values of the three components with TYR, TYRP-1 and DCT were all below −7.0 kcal/mol, indicating strong binding activity [24]. In conclusion, the above data suggested that the three components could bind TYR, MITF, TYRP-1 and DCT and modulate their functions. In addition, TYR, TYRP-1 and DCT may be critical targets for the whitening effects of these three components.

The results of molecular docking with the lowest protein binding energy for each core target were visualized using PyMol 4.5.0 software, and the three-dimensional docking diagrams of the three compounds with TYR, TYRP-1 and DCT were shown in Figure 7B–D. The results showed that the three components generated a large number of hydrogen bonds and van der Waals forces with the inhibitory sites of TYR, TYRP-1, and DCT, as well as hydrophobic effects such as π-alkylation, which reduced the entropy of the system and made the system more stable. Hydrogen bonding is the main force that facilitates the binding of the ligand to the active site [25]. Resveratrol binds to the TYR active sites Gly103 and Gln105 to form hydrogen bonds and binds to TYRP-1 by forming hydrogen bonds with GLY461, PRO242, GLN236 and TYR226 (green line). Resveratrol is predicted to bind to DCT by forming hydrogen bonds with GLN32 TYR64 and TYR27 (Figure 7B). As shown in Figure 7C, emodin is predicted by docking simulation to bind to the active site of TYR by forming hydrogen bonds with TYR35 (green line). Emodin is predicted by docking simulation to bind to the active site of TYRP-1 by forming hydrogen bonds with ASN429 and THR176 (green line). Emodin is predicted by docking simulation to bind to the active site of DCT by forming hydrogen bonds with TYR68, ASP30, GLY237 and GLN32 (green line). As shown in Figure 7D, Physcion may bind to TYR as well through forming hydrogen bonds with ARG6, ASP30 and TYR27 in the DNA binding site of TYR (green line). Physcion is predicted by docking simulation to bind to the active site of TYRP-1 through forming hydrogen bonds with GLN236, THR112, LYS233, CYS113, ARGC118 and ARG114 (green line). Physcion is predicted by docking simulation to bind to the active site of DCT through forming hydrogen bonds with GLN32, SER53, TYR68 and ARG48 (green line). Interestingly, resveratrol, emodin and physcion could form hydrophobic bonds (pink line) to the same catalytically active amino acid residues (PRO235) of DCT and could form hydrogen bonds (green line) to the same catalytically active amino acid residues (GLN32) of DCT, which may be one of the reasons why these compounds have anti-melanogenesis effects.

#### 2.5.2. Down-Regulation of Both Transcripts of Melanogenesis-Related Factors

**Figure 8:**
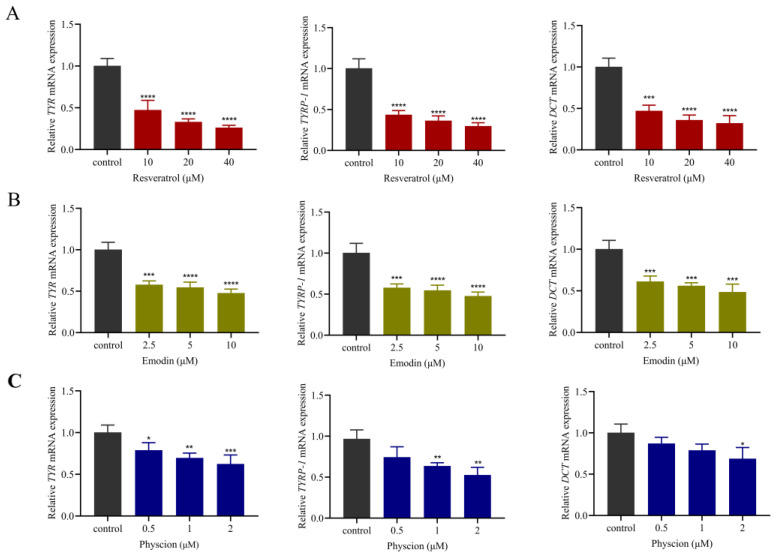
Effects of three components (resveratrol, emodin and physcion) on the expression of melanogenesis-related factors in B16F10 cells. The relative quantification of mRNA expression in B16F10 cells was performed by qRT-PCR. (**A**) Relative TYR, TYRP-1, DCT mRNA expression after resveratrol treatment. (**B**) Relative TYR, TYRP-1, DCT mRNA expression after emodin treatment. (**C**) Relative TYR, TYRP-1, DCT mRNA expression after physcion treatment. (Data are expressed as the means ± SD of three independent experiments. * *p* < 0.05, ** *p* < 0.01, *** *p* < 0.001 and **** *p* < 0.0001 compared with the control using the one-way ANOVA comparison test.)

It is well known that melanogenesis involves the regulation of several melanogenesis-related proteins, such as TYR, TYRP-1, and DCT [26]. Therefore, based on molecular docking, to elucidate the melanogenesis inhibition mechanism of resveratrol, emodin and physcion, we detected the gene transcription of several factors with strong binding ability to the predicted active compounds in B16F10 cells by qRT-PCR assay. As shown in Figure 8, the transcript levels of TYR, TYRP-1 and DCT could be significantly suppressed after we treated B16F10 cells with resveratrol, emodin and physcion. Overall, these data suggested that these three predicted active components inhibited melanogenesis by down-regulating the expression of melanogenesis-related factors at the mRNA level.

## 3. Discussion

Natural extracts from traditional Chinese medicine have the characteristics of multicomponents, multi-targets and multi-functions, which can inhibit tyrosinase activity during melanin synthesis, reduce melanin transporter, and accelerate skin metabolism [27,28]. Whitening products are an important part of cosmetics and herbal active ingredients have great potential in the whitening market. From the consumer’s perspective, natural products are more skin-friendly, so herbal active ingredients are more readily accepted, thus contributing to the tremendous progress in herbal medicine research.

PC extract has been widely used in cosmetic products and is rich in resveratrol, a compound that has been hotly researched in recent years due to its tyrosinase inhibition, anti-aging and moisturizing effects [29]. Our study found that the melanogenesis inhibition of PC extract sold by different manufacturers varies to some extent, which may be due to differences in the content of active ingredients. Some ingredients such as stilbenes, flavonoids and anthraquinones have been reported in the literature to be able to inhibit melanogenesis [15,16]. Melanocytes have a series of mechanisms to regulate melanin synthesis, among which the “triple enzyme theory” of melanin synthesis initiation is widely accepted. The “triple enzyme” refers to TYR, TYRP-1 and DCT, of which TYR is the key enzyme that coordinates melanin production [1]. Therefore, we examined the melanogenesis inhibition of PC extract from various batches using the inhibition rate of TYR in B16F10 cells as the index. At the same time, we investigated the material basis of the whitening of PC extract by spectrum–effect relationship and explored the mechanism of melanogenesis inhibition.

A statistical analytic technique called the spectrum-effect relationship is utilized to link the characteristics of traditional Chinese medicines to their efficacy, which is frequently employed in the active ingredient screening of conventional Chinese medications. Currently, the statistical analysis methods used in the spectrum-effect correlation method include correlation analysis, regression analysis, principal component analysis, gray correlation analysis, and artificial neural network analysis. Each statistical method has its advantages and disadvantages, so the most reasonable and effective prediction can be obtained by combining multiple results in practical application [30,31,32]. In order to screen the active ingredients, we used the spectrum-effect relationship method, combining 11 common peaks and pharmacological parameters of PC extract using Pearson’s correlation analysis, GRA and OPLSR. Our study found that P7 (resveratrol), P10 (emodin) and P11 (physcion) were the most important active ingredients with whitening effects.

To further explore the whitening mechanism of the active ingredients, we found from molecular docking that P7, P11 and P12 all bind to TYR, MITF, TYRP-1 and DCT, and form stable complexes. At the same time, the three components were able to bind to TYR, TYRP-1 and DCT with binding energies <−7 kcal/mol, suggesting that strong binding to the targets could be formed thereby modulating their effects. The 3D and 2D diagrams of the molecular docking showed that the three components generated a large number of hydrogen bonds and van der Waals forces with the inhibitory sites of TYR, TYRP-1, and DCT, as well as hydrophobic effects such as π-alkylation, which reduced the entropy of the system and made the system more stable.

In summary, this study found that resveratrol, emodin and physcion may directly inhibit TYR. Therefore, resveratrol, emodin and physcion will be used in future quality control. This study demonstrated that the spectrum-effect relationship could be used as an effective method to find active ingredients in complex compound systems. Meanwhile, this study also explored the material basis of PC extract to inhibit melanogenesis, which provided theoretical support for the subsequent quality control of cosmetic raw materials.

## 4. Materials and Methods

### 4.1. Materials and Reagents

The source information of 10 batches of PC extract used for the experiment is shown in Table 4. Reference substances (purity ≥ 98%) including resveratrol, quercetin, emodin, physcion and emodin-8-β-D-glucoside were purchased from the National Institute for the Control of Pharmaceutical and Biological Products (NICPBP). Dimethyl sulfoxide (DMSO) was purchased from Beijing Solarbio Science & Technology Co., Ltd. (Beijing, China). TritonX-100 was bought from GBCBIO Technologies lnc. (Guangzhou, China). L-DOPA was purchased from Yingxin Laboratories (Shanghai, China). Trypsin, penicillin-streptomycin solution and DMEM were purchased from Cytiva (Marlborough, MA, USA). Fetal bovine serum (FBS) was bought from Zhejiang Tianhang Biological Technology Co. (Huzhou, China). The murine melanoma cell line B16F10 (C57BL/6) and RPMI-1640 complete medium were obtained from Procell Life Science & Technology Co., Ltd. (Wuhan, China). The SweScript RT II First Strand cDNA Synthesis Kit and 2xUniversal SYBR Green qPCR Master Mix were purchased from Wuhan Servicebio Technology Co., Ltd. (Wuhan, China).

### 4.2. HPLC Fingerprint

#### 4.2.1. Establishment of HPLC Condition

Chromatographic separation was performed on an Agilent 1200 HPLC system with a ZORBAX Eclipse XDB-C18 column (4.6 mm × 250 mm, 5 μm) (Thermo Scientific TM, Waltham, MA, USA). The mobile phase was a mixture of acetonitrile (A) and 0.2% phosphoric acid water (B). The injection volume was 10 μL, and the flow rate was 1.0 mL/min. The detection wavelength and column temperature were set at 290 nm and 40 °C, respectively. The optimized gradient elution steps were: 0–10 min: 10–24% A, 10–12 min: 24–25% A, 12–15 min: 25–28% A, 15–25 min: 28–30% A, 25–35 min: 30–31% A, 35–40 min: 31–35% A, 40–50 min: 35–90% A, and 50–60 min: 90–100% A. All sample solutions were filtered with the 0.45 μm microporous membrane before injection.

#### 4.2.2. Preparation of Sample Solutions

For the HPLC analysis, 0.1 g of each extract was precisely weighed and ultrasonically extracted in 25 mL of methanol for 30 min. The filtrate was filtered through a 0.45 μm microporous membrane and stored in a refrigerator at 4 °C for analysis. All samples underwent the aforementioned processing.

#### 4.2.3. Preparation of Standard Solutions

Three standard compounds (resveratrol, emodin and physcion) were prepared into a storage solution at concentrations of 0.19, 0.19, and 0.21 mg/mL. Then, filtered with 0.45 μm microporous membrane after constant volume, and finally sealed and stored in a dark place at 4 °C as a reserve solution for backup.

#### 4.2.4. Validation of the Fingerprint Analysis Process

Fingerprint analysis method validation was performed by determining the relative standard deviation (RSD) value of relative retention time (RRT) and the relative peak area (RPA) of PC extract, respectively. According to the literature method [33], the RSD values of the same sample after successive injections, repeated injections, and injections at different time points were evaluated for precision, repeatability and stability.

#### 4.2.5. Similarity Analysis of Fingerprint

Under the above HPLC condition, the representative fingerprints of 10 batches of PC extract were established, and their similarity was analyzed based on the Similarity Evaluation System for Chromatographic Fingerprint of the Chinese Medicine (2012 Version).

### 4.3. Pharmacodynamic Activity

#### 4.3.1. Cell Culture

The mouse melanoma cell line B16F10 (C57BL/6) was cultured in RPMI 1640 complete medium and incubated in a humidified incubator at 37 °C with 5% CO_2_.

#### 4.3.2. Cytotoxicity Analysis

The cytotoxicity of PC extract to B16F10 cells was evaluated using a CCK-8 assay method. Briefly, 1 × 10^4^ cells/well were seeded in 96-well plates and incubated overnight. After discarding the supernatant, the cells were treated with different concentrations of PC extract for 48 h. Afterward, 10 μL of CCK-8 reagent was added to each well and the cells were incubated for another hour. The optical density (OD) of each well was measured at 450 nm by a microplate reader (xMark^TM^, Bio-Rad, Benicia, CA, USA). The percentage of surviving cells was calculated according to the following equation:

Cell viability (%) = (OD_sample_ − OD_blank_)/(OD_control_ − OD_blank_) × 100

OD_sample_, OD_blank_ and OD_control_ represent the absorption at 450 nm for cells treated with different concentrations of PC extract, blank medium, and normal cells without drugs, respectively.

#### 4.3.3. Intracellular TYR Inhibition Rate Assay

TYR activity in B16F10 cells was detected according to the previous reports [26,34]. B16F10 cells at logarithmic growth period were inoculated in a 96-well culture plate with a density of 1.0 × 10^4^ cells per well for 24 h. Cells were then incubated with different concentrates of PC extract for 48 h. After treatment, the cells were washed twice with PBS (pH = 6.8), incubated with 100 μL of TritonX-100 solution, and frozen at −80 °C for 1 h. After the cells were thawed and lysed at room temperature, 0.1% L-DOPA 100 μL was added to each well sequentially, and then the cells were incubated at 37 °C for 2 h. The OD value of each well at 475 nm was determined by a microplate reader (xMarkTM, Bio-Rad, USA).

### 4.4. Spectrum-Effect Relationships Analysis

#### 4.4.1. Pearson’s Correlation Analysis

The type and degree of the relationship between the two variables can be determined by using Pearson’s correlation analysis to predict and explain the linear relationship between the two variables [35]. Pearson’s correlation analysis was performed by using IBM SPSS Statistics 26 software. The peak area of the shared peaks of PC extract was regarded as the independent variable (X), and the TYR inhibition rate of the administered groups was regarded as the dependent variable (Y) [36].

#### 4.4.2. Gray Relational Analysis (GRA)

GRA is a widely utilized mathematical research technique that can identify the connection between the chemical substances and the effectiveness of Traditional Chinese Medicine (TCM), to screen effective active TCM compounds [37,38]. In this article, the correlation between the common peaks of the 10 batches of PC extract and their pairs of intracellular TYR contents according to the GRA method.

#### 4.4.3. Orthogonal Partial Least Squares Regression Analysis (OPLSR)

For OPLSR, the common peak areas were set as independent variables (X) and the biological activity levels of different batches were regarded as dependent variables (Y). SIMCA 14.1 software was used to establish the OPLSR model. The regression coefficient was regarded as an index that exhibited the relative impact of the independent variables on the dependent variables. The VIP value was used to identify chromatographic peaks significantly correlated with the biological activities of the extracts.

### 4.5. Verifications of Predicted Active Substances’ In Vitro Activity

#### 4.5.1. Intracellular TYR Inhibition Assay

TYR activity in B16F10 cells after incubation with different predicted active substances from PC extract was assayed as described in Section 4.3.3 of this article.

#### 4.5.2. Measurement of Melanin Level

The quantity of melanin in B16F10 cells was assessed as the previous report [26]. B16F10 cells were inoculated with DMEM cell culture medium containing 10% fetal bovine serum (FBS), penicillin 100 U/mL and streptomycin 100 U/mL at a density of 2 × 10^5^ cells/well in 6-well plates overnight, and then treated with different concentrations of resveratrol, rhodopsin and paracetamol for 48 h. After treatment, the medium was discarded and the cells were collected and resuspended in 500 μL of 1 M NaOH aqueous solution containing 10% DMSO. The samples were heated in a water bath at 80 °C for 1 h and then vortexed until the melanin from the cells was completely dispersed and dissolved. After centrifugation at a speed of 3000 r/min for 5 min, 100 μL of the supernatant was placed in a 96-well plate to measure the OD value at 405 nm by a microplate reader.

### 4.6. Compound-Target Molecular Modeling

The strategy of molecular docking was adopted to explore the interaction and affinity between the active compounds screened through the spectrum–effect relationship and key targets. The structures of all compounds were derived from the PubChem database (https://pubchem.ncbi.nlm.nih.gov) (accessed on 12 September 2023), and the crystal structure of the target proteins was obtained from PDB (https://www.rcsb.org) (accessed on 12 September 2023) [39]. The three predicted whitening active ingredients were used as ligand molecules, and MITF, TYR, TYRP-1, DCT were used as receptor proteins, and the water molecules, ligands and small molecules in the inactive parts of the crystal structures were removed to obtain the receptor proteins for molecular docking. The molecular docking was performed using AutoDock Tools 1.5.6 software, and the molecular docking results were visualized using Discovery Studio Visualizer v21.software.

### 4.7. Gene Expression Analysis by qRT-PCR

qRT-PCR was used to determine the transcription levels of TYR, TYRP-1, and DCT in B16F10 cells treated with resveratrol, emdion, and physcion, respectively. The total RNA was recovered from B16F10 cells after chemical treatment and reverse-transcribed into first-stranded cDNA using the SweScript RT II First Strand cDNA Synthesis Kit (Servicebio, China). Then, following the manufacturer’s recommendations, cDNAs were used for real-time PCR analysis in a CFX96 Real-Time PCR system (Bio-Rad, USA) using 2×Universal SYBR Green qPCR Master Mix (Servicebio, China). Table 5 displays the real-time PCR primer sequences. Data were evaluated using the previously mentioned 2^−ΔΔCt^ method after being normalized to the GAPDH expression levels [40,41].

### 4.8. Statistical Analysis

All the results were expressed as the mean ± SD. The unpaired two-tailed student’s *t*-test was performed for pairwise comparisons and one-way ANOVA was performed for comparisons of more than two groups. *p* < 0.05 was considered significant. All statistical tests were performed by GraphPad Prism 8 statistical software. IBM SPSS Statistics 26 was used for Pearson’s correlation analysis. SIMCA 14.1 version was used for orthogonal partial least-squares analysis (OPLSR).

## 5. Conclusions

In this study, we established a spectrum–effect relationship for the inhibition of TYR activity by PC extracts and screened PC extracts for anti-melanogenesis actives. In vitro cellular experiments verified our predictions that resveratrol, emodin, and physcion have a whitening effect. We also revealed the interactions between the active small molecules and the protein target sites using the molecular docking technique. In addition, the down-regulatory effects of the anti-melanogenesis active ingredients on the transcriptional levels of key targets were verified by qRT-PCR. This study not only elucidated the material basis closely related to the whitening efficacy of PC extract but also contributed to the extensive and rational application of PC extract in cosmetics and facilitated the pharmacological study and quality control of PC extract in the future.

## Figures and Tables

**Table 1:** Results of precision, repeatability and stability of relative retention times and relative peak areas.

Peak NO.	RRT-RSD (%)			RPA-RSD (%)		
	Precision	Repeatability	Stability	Precision	Repeatability	Stability
1	0.03%	0.08%	0.09%	0.29%	0.71%	1.06%
2	0.02%	0.06%	0.09%	0.62%	1.34%	1.30%
3	0.02%	0.04%	0.06%	0.31%	1.23%	1.09%
4	0.02%	0.01%	0.03%	0.10%	2.17%	2.07%
5	0.01%	0.02%	0.04%	0.09%	1.82%	2.32%
6	0.03%	0.01%	0.02%	0.60%	1.55%	1.59%
7	0	0	0	0	0	0
8	0.02%	0.01%	0.01%	0.46%	1.17%	1.10%
9	0.04%	0.01%	0.03%	0.36%	1.13%	0.53%
10	0.03%	0.03%	0.01%	0.28%	1.40%	1.17%
11	0.03%	0.03%	0.01%	0.37%	1.32%	1.01%

**Table 2:** Results of Pearson’s correlation analysis between each common peak and tyrosinase inhibition.

Peak	Correlation Coefficient	*p* Value
P1	−0.628	0.052
P2	0.153	0.673
P3	−0.462	0.179
P4	−0.250	0.485
P5	−0.097	0.790
P6	−0.393	0.262
P7	0.739 *	0.015
P8	0.162	0.656
P9	0.427	0.218
P10	0.704 *	0.023
P11	0.675 *	0.032

“*” indicates the correlation is significant at the 0.05 level.

**Table 3:** Gray correlation and ranking results of each common peak.

Peak	Correlation Degree	Ranking
P7	0.917	1
P8	0.826	2
P4	0.810	3
P5	0.805	4
P10	0.802	5
P2	0.779	6
P11	0.776	7
P9	0.740	8
P1	0.736	9
P3	0.680	10
P6	0.577	11

**Table 4:** Origin of the 10 batches of *Polygonum cuspidatum* extract.

No.	Origin	Lot. NO	Resveratrol Content
S1	Hubei Yitai Biotechnology Co.	221104	≥50%
S2	Hubei Yitai Biotechnology Co.	221113	≥50%
S3	Shanxi MuMeng Biotechnology Co.	221108	50%
S4	Dongming Growth Biotechnology Co.	221202	≥50%
S5	Dongming Growth Biotechnology Co.	221206	≥50%
S6	Shanxi Ducheng Pharmaceutical Technology Co.	220910	≥50%
S7	Shanxi Baicao Xintian Biotechnology Co.	221018	50%
S8	Hubei Province Sanxin Biotechnology Co.	221121	≥50%
S9	Hubei Province Sanxin Biotechnology Co.	221123	≥50%
S10	Hubei Province Sanxin Biotechnology Co.	221127	≥50%

**Table 5:** Sequences of the primers used for quantitative real-time PCR.

Gene Name	Direction	Sequence
MITF	Forward	TGAAGCAAGAGCATTGGCTA
	Reverse	TCCACAGAGGCCTTGAGAAT
TYR	Forward	CCTCCTGGCAGATCATTTGT
	Reverse	GGTTTTGGCTTTGTCATGGT
TRP-1	Forward	CTGCGATGTCTGCACTGA TGACTTG
	Reverse	TTTCTCCTGATTGGTCCACCCTCAG
TRP-2	Forward	GGATGACCGTGAGCAATGGCC
	Reverse	CGGTTGTGACCAATGGGTGCC
GAPDH	Forward	GGCAAATTCAACGGCACAGTCAA
	Reverse	GACATACTCAGCACCGGCCTCAC

## Data Availability

Data are contained within this paper.

## Abbreviations

PC	*Polygonum cuspidatum*	TYR	tyrosinase
HPLC	high-performance liquid chromatography	TYRP-1	tyrosinase-related protein 1
GRA	gray correlation analysis	DCT	dopachrome tautomerase
CCK-8	cell counting kit-8	L-DOPA	levodopa
OPLSR	orthogonal partial least squares regression analysis	MITF	melanocyte-inducing transcription factor
PCR	polymerase chain reaction	TCM	traditional Chinese medicines
RSD	relative standard deviation	RPA	relative peak area
DMSO	dimethyl sulfoxide	FBS	fetal bovine serum
OD	optical density	RRT	relative retention time
cDNA	complementary DNA	VIP	very important person
UV	ultraviolet		
